# Odontoid process type II and III fracture fixation using bone allograft screws versus cannulated screws: a biomechanical study

**DOI:** 10.1007/s00402-025-05805-z

**Published:** 2025-03-22

**Authors:** Emir Benca, Kenneth P. van Knegsel, Maximilian Pestel, Ivan Zderic, Jan Caspar, Lena Hirtler, Andreas Strassl, Dominic Gehweiler, Sonja Zehetmayer, Boyko Gueorguiev, Harald Widhalm, Reinhard Windhager, Peter Varga

**Affiliations:** 1 Department of Orthopedics and Trauma Surgery, Medical University of Vienna, Währinger Gürtel 18- 20, Vienna, 1090 Austria; 2 AO Research Institute Davos, Clavadelerstrasse 8, Davos, 7270 Switzerland; 3 Department of Orthopedics and Trauma Surgery, Cantonal Hospital of Lucerne, Spitalstrasse 16, Lucerne, 6000 Switzerland; 4 Faculty of Medicine, University of Zurich, Raemistrasse 100, Zurich, 8091 Switzerland; 5 Division of Anatomy, Center for Anatomy and Cell Biology, Medical University of Vienna, Währinger Strasse 13, Vienna, 1090 Austria; 6 Department of Biomedical Imaging and Image-Guided Therapy, Medical University of Vienna, Währinger Gürtel 18-20, Vienna, 1090 Austria; 7 Center for Medical Data Science, Medical University of Vienna, Spitalgasse 23, Vienna, 1090 Austria

**Keywords:** C2 vertebra, Odontoid process fracture, Bone allograft screw, Cannulated screw, Biomechanics

## Abstract

**Introduction:**

Fractures of the odontoid process are associated with high non-union rates, challenging treatment, and high incidence of screw-related complications. The aim of this study was to compare the biomechanical competence of a single biointegrative bone allograft screw versus two conventional cannulated screws for odontoid fracture fixation.

**Materials and methods:**

The odontoid process of intact C2 vertebral specimens was subjected to quasi-static loading until fracture. Specimens with an Anderson and d’Alonzo type II or III fracture (*n* = 47) were fixated with either two conventional cannulated screws or with a single bone allograft screw. The constructs were biomechanically tested to failure in the same fashion as in their intact state. Stiffness, yield, and ultimate load were evaluated. The results were adjusted by age, sex, volumetric bone mineral density (vBMD), and the cross-sectional area ratio of cortical bone to total bone measured at the junction of the odontoid process with the vertebral body (Ct.Ar/Tt.Ar).

**Results:**

Stiffness, yield and ultimate load were restored in the cannulated screws group by 44 ± 10%, 46 ± 7%, and 46 ± 5% and in the bone allograft group by 50 ± 12%, 30 ± 9%, and 34 ± 6% (mean ± SE). There were no significant differences between the groups regarding the three mechanical outcomes (0.104 ≤ *p* ≤ 0.223). Positive significant relation was found between vBMD and stiffness in each group (0.248 ≤ R²≤0.273, 0.018 ≤ *p* ≤ 0.038), as well as between Ct.Ar/Tt.Ar and stiffness (R²=0.218, *p* = 0.033), vBMD and ultimate load (R²=0.430, *p* = 0.001) and ultimate loadand vBMD (R²=0.315, *p* = 0.010) in the cannulated screws group.

**Conclusions:**

The primary stability of odontoid fracture fixation is determined mainly by the quality of the local bone and independent of the fixation technique. From the biomechanical perspective, the lower mean values for the yield and ultimate load restored in the bone allograft group compared to the cannulated screws group should be compensated by lower peak load during the patient’s rehabilitation process.

## Introduction

Odontoid fractures are among the most common spinal fractures in both the general and in the elderly populations. In the latter, they represent even the most common isolated cervical spine fracture [1]. These fractures are classified into three types according to Anderson and D’Alonzo [2]. Whereas type I is an oblique fracture through the superior part of the odontoid process, type II is a fracture at the junction of the odontoid process with the vertebral body, and type III is a fracture through the anterior body of the C2. The average age of patients suffering an odontoid fracture is 58 years for the general population [3], and 82 years for the elderly ≥ 65 years [4]. While type I fractures are rarely observed and do not require surgical treatment, type II and III fractures are frequent and often indicated for surgical treatment, especially in case of instability. The average age of patients who undergo surgery is 53 years, with 84% of them having a type II and 8% having a type III fracture [5]. The main goal of both conservative and surgical treatments is to avoid atlantoaxial instability, which can lead to neurological injuries resulting in severe complications and death [3]. Overall, 40% of the elderly patients with odontoid type II fractures are treated surgically [6]. Surgical fixation with metal screws has been the most widely used method of odontoid fracture fixation [5] performed by means of single-screw or double-screw fixation using either double-threaded, fully-threaded or lag screws inserted through the anterior-inferior lip of the C2 body or its lower endplate. However, despite its wide utilization, odontoid screw fixation is associated with a high risk of screw-related complications including fracture non-union (point estimation of incidence: 9.7%), screw cut-out (5.0%), screw malposition (4.8%), screw loosening (3.8%), and screw breakage (3.1%) [5]. The non-union rate is significantly correlated with patient age [5]. Among older patients (≥ 65 years), screw cut-out occurs significantly more often following double-screw compared to single-screw fixation, while in the younger population (< 65 years) the cut-out rate is independent of the number of inserted screws [5].

Given the high incidence of odontoid fractures and the high non-union rates as well as other implant-related complications, especially in the elderly, the current surgical management of odontoid fractures still has potential for improvement. Using bone allografts could overcome some clinical problems, such as fracture non-unions. The bone allograft screw Shark Screw (surgebright GmbH, Lichtenberg bei Linz, Austria) is a threaded cylinder milled from human cortical bone. It has been previously used in hand [7, 8] and foot surgery [9] with good short- and long-term outcomes. A case study reported after ten weeks following the first metatarsophalangeal joint arthrodesis surgery, the vascularization of the graft’s Haversian pore network including osteoclastic and osteoblastic activity, creating new bone at the bone-graft interface [10]. Along with no reported immunological rejections and no potential imaging artefacts as experienced from metal devices, the use of this bone allograft screw is potentially feasible in the management of type II and III odontoid process fractures. However, prior to the clinical application, its biomechanical performance in restoring the primary stability has to be evaluated.

Therefore, the aim of this study was to quantify the biomechanical competence of a single bone allograft screw to restore the physiological stability (in terms of stiffness, yield., and ultimate load) of the odontoid process following type II and III fractures in comparison to the gold standard of double-screw fixation [11] under consideration of both the full physiological range of loading and bone quality. The study hypothesis was that the bone allograft screw could restore the intact stability to the same level as the pair of cannulated screws.

## Materials and methods

**Fig. 1:**
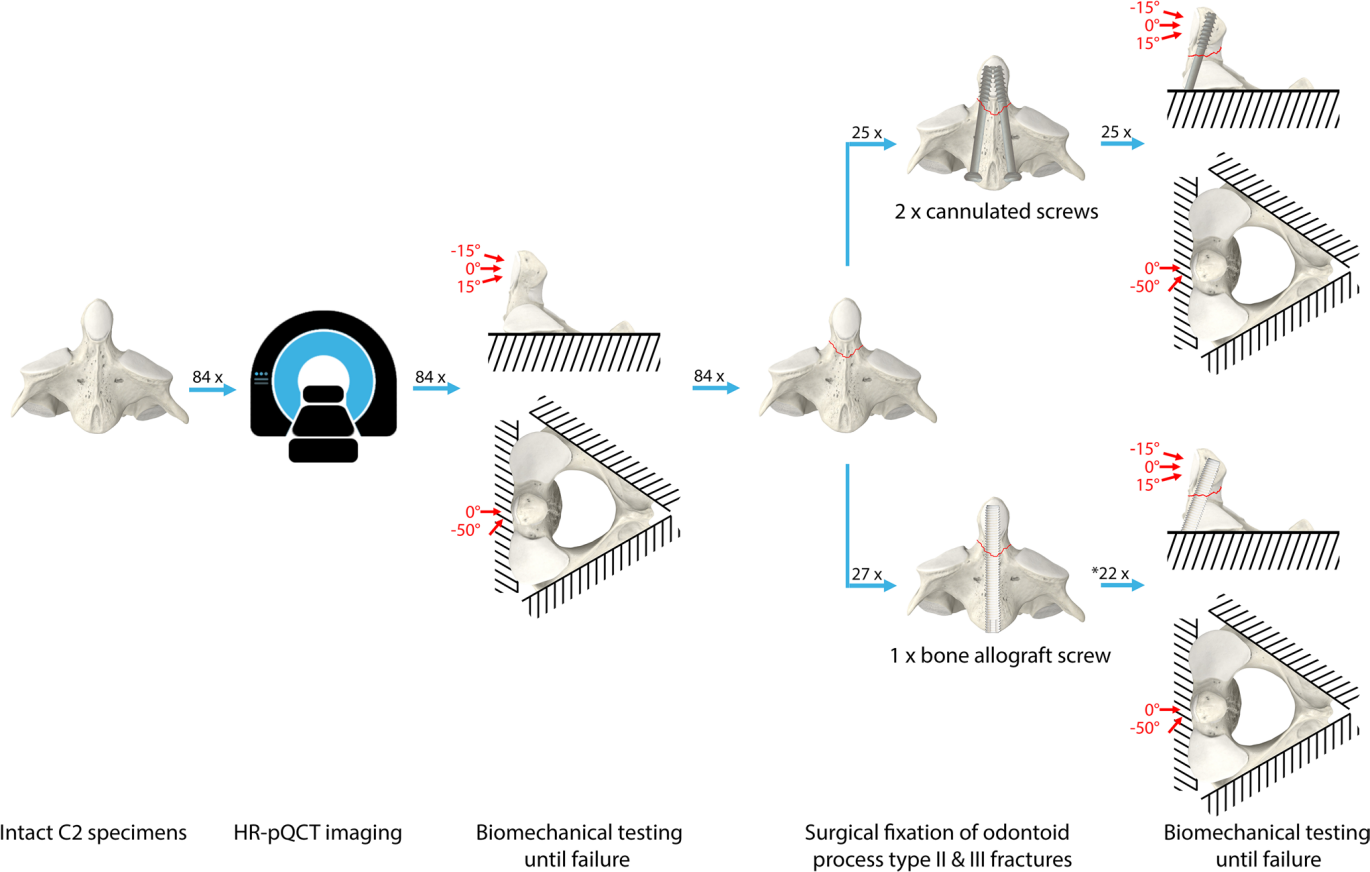
An overview of the study methodology. Eighty-four C2 specimens were scanned in high-resolution peripheral quantitative computed tomography (HR-pQCT) and biomechanically tested in physiological range of load orientation until fracture. All specimens with an Anderson and d’Alonzo type II and III fractures were surgically fixated with two cannulated screws or with a single bone allograft screw (*five specimens in this group were used for pilot testing and were, thus, excluded from the study). Fixation constructs were then biomechanically tested to failure in the same manner as in their intact state

Isolated C2 specimens with biomechanically induced type II and III odontoid fractures were randomized for treatment with either two cannulated metal screws or a single bone allograft screw and biomechanically tested to failure under various loading conditions. An overview of the methodology is illustrated in Fig. 1.

### Specimens

Eighty-four anatomic specimens of the human axis (second cervical vertebra (C2)) from adult body donors (age > 18 years) were collected at the Medical University of Vienna. The donors provided informed consent about body donation during their lifetime and the study was approved by the Ethics Committee of the Medical University of Vienna (2141/2020) and conforms to the Declaration of Helsinki. All specimens were inspected for tendon degeneration or previous surgical treatment. The specimens were thawed at room temperature for approximately 24 h and kept moist with 0.9% saline solution during the preparation and biomechanically tested at room temperature. Following stripping of soft tissues, the specimens were embedded in aluminum cylinders using polymethylmethacrylate (PMMA, SCS-Beracryl D28, Suter Kunststoffe AG, Fraubrunnen, Switzerland) up to the level of the superior articular surface. The alignment ensured that the axis of the odontoid process coincided with the cylinder axis using line laser beams. The anterior part of the inferior C2 was excluded from the embedding in order to allow screw insertion after surgical reduction.

The specimens were assigned to six groups of 14 specimens each, designed to include the full physiological range of loading directions to the odontoid process during biomechanical testing (see Biomechanical Testing and Data Evaluation). The fracture load and pattern results for 42 specimens have been previously published [12]. It has to be noted, that five out of 27 specimens with type II or type III fractured were used for pilot testing and thus, were not included in the current study resulting in 22 specimens in the bone allograft group. Another 42 specimens were tested to fracture in this study. Following biomechanical testing, only specimens with Anderson and d’Alonzo type II and III fracture [2] (*n* = 47) were further included in the fixation stability study (Table 1).

**Table 1:** Characteristics of the specimens with Anderson D’Alonzo type II and III fractures, sorted based on the loading direction and fixation type. The data for each group are presented as (MIN - MAX) and pooled data– as mean ± sd. Abbreviations: vertical load angle (VLA), horizontal load angle (HLA)

		Cannulated screws				Bone allograft screw			
HLA	VLA	n	Sex (f/m)	Age (years)	Fracture type	n	Sex (f/m)	Age (years)	Fracture type
0°	0°	3	2f, 1 m	(71–81)	II: 2, III: 1	4	2f, 2 m	(69–74)	II: 2, III: 2
-50°	0°	6	1f, 5 m	(79–101)	II: 3, III: 3	5	1f, 4 m	(63–78)	II: 5, III: 0
0°	-15°	5	5f, 0 m	(65–93)	II: 3, III: 2	6	0f, 6 m	(59–76)	II: 1, III: 5
-50°	-15°	4	2f, 2 m	(78–90)	II: 2, III: 2	2	1f, 1 m	(68–79)	II: 0, III: 2
0°	15°	2	0f, 2 m	(75–83)	II: 0, III: 2	2	1f, 1 m	(77–78)	II: 1, III: 1
-50°	15°	5*	5f, 0 m	(71–95)	II: 3, III: 2	3	1f, 2 m	(65–76)	II: 1, III: 2
Total		25	15f, 10 m	82.5 ± 8.4 (65–101)	II: 13, III: 12	22	6f, 16 m	71.2 ± 5.6 (59–79)	II: 10, III: 12

*one specimen was excluded due to incorrect alignment during biomechanical testing

### HR-pQCT scanning and image analysis

Densitometric and geometrical variables were determined utilizing high-resolution peripheral quantitative computed tomography (HR-pQCT, XtremeCT, Scanco Medical AG, Bassersdorf, Switzerland) scanning at 60 kVp, 900 µA, 750 projections, 200 ms acquisition time, and 82 μm resolution. All specimens were scanned axially and in their intact state.

**Fig. 2:**
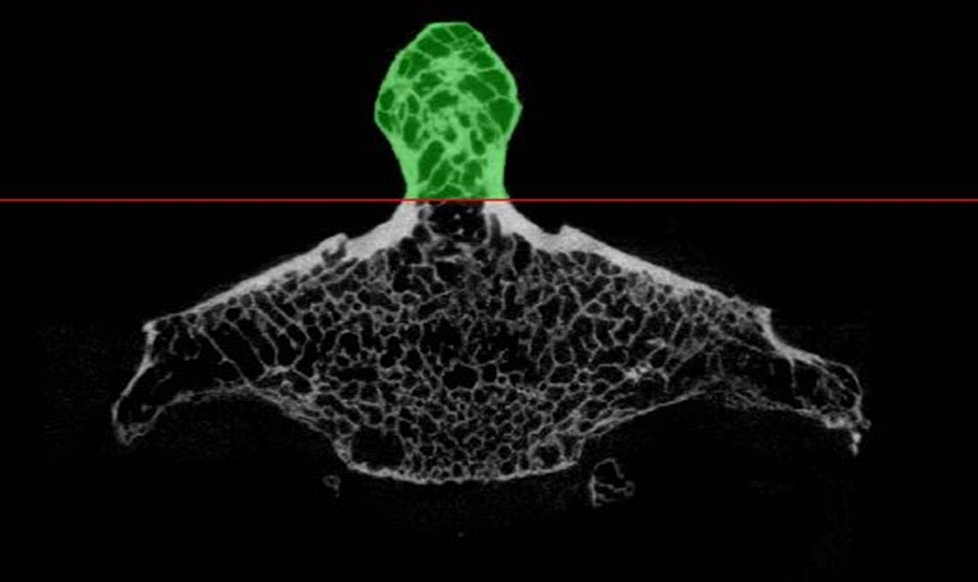
Region of interest for the assessment of the volumetric bone mineral density (vBMD) (green). The cross-sectional ratio of cortical bone to total bone (Ct.Ar/Tt.Ar) was assessed in the most caudal CT slice indicated by the red line

The volumetric bone mineral density (vBMD) of the isolated odontoid process and the cross-sectional ratio of cortical bone to total bone measured at the junction of the odontoid process with the vertebral body, were evaluated (Materialise Mimics V.22.0 and V.24.0; Materialise NV, Leuven, Belgium) as follows. The CT images were rotated to align the odontoid process axis with a vertical axis in the coronal image plane. Next, a mask was created combining automatic and manual segmentation tools including global thresholding with a vBMD value of 290 HA mg/cm^3^, automatic infill and manual brush tools, to isolate the odontoid process for further analysis. The mask was standardized such that its caudal margin was set in the axial cross-section where the contour odontoid process forked into the transition to the vertebral body (Fig. 2). vBMD was then evaluated within the masked region.

**Fig. 3:**
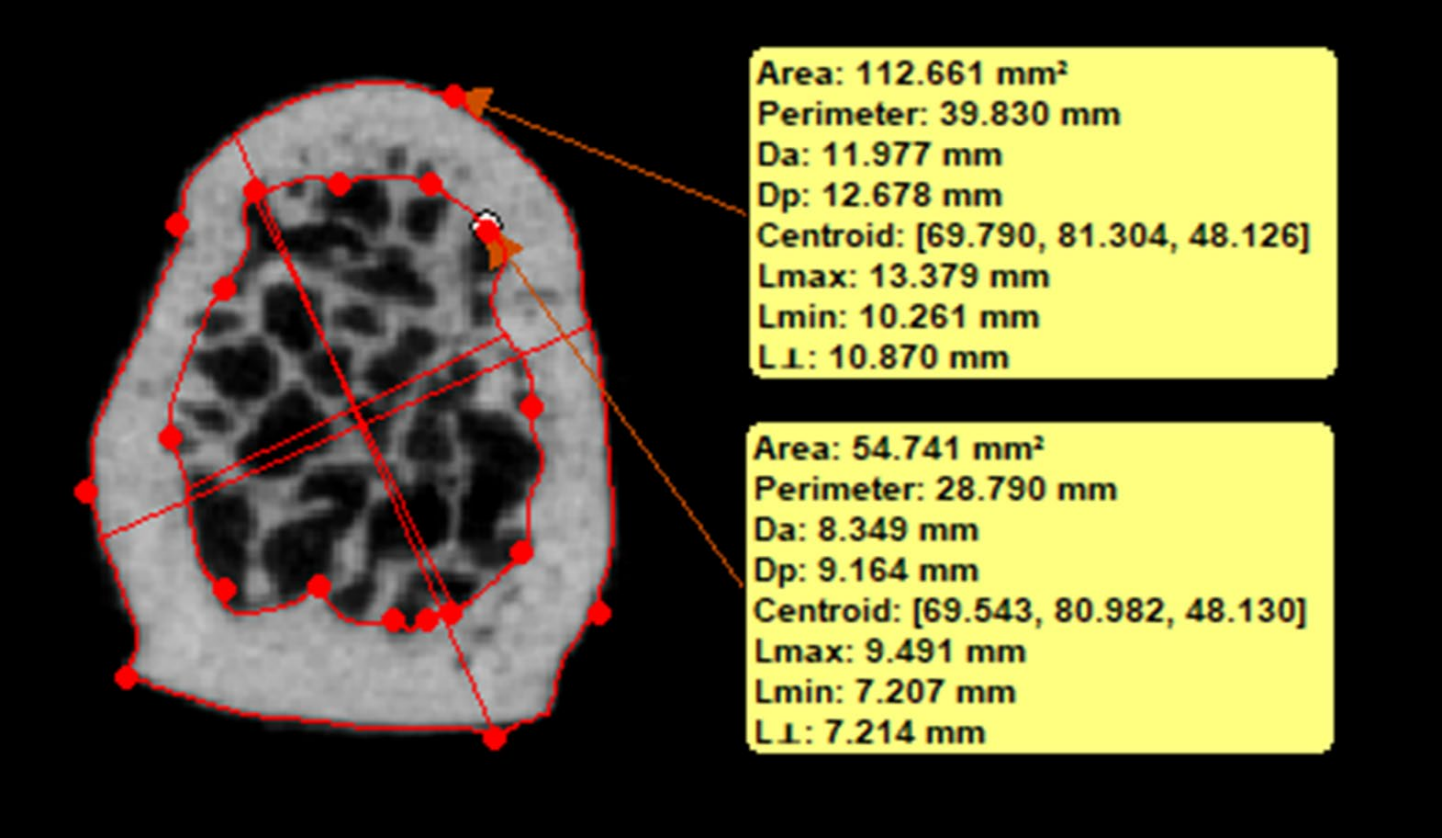
Assessment of specimen-specific cross-sectional area and ratio of cortical bone of the odontoid process. Two polylines were created based on multiple manually set points on the outer and inner contour of the cortical bone

The most caudal axial section of the mask was further analyzed to determine the total area of the odontoid process (Tt.Ar) and the area of the cortical bone (Ct.Ar) to calculate the cross-sectional ratio of cortical bone to total bone (CT.Ar/Tt.Ar). This was achieved by manually defining two polylines on the outer and inner contour of the cortical bone (Fig. 3).

The HR-pQCT data were corrupt for three specimens of the bone allograft screw group and the assessment of the cortical ratio was not feasible for one specimen of the cannulated screws group due to the presence of large cavities.

### Biomechanical testing and data evaluation

**Fig. 4:**
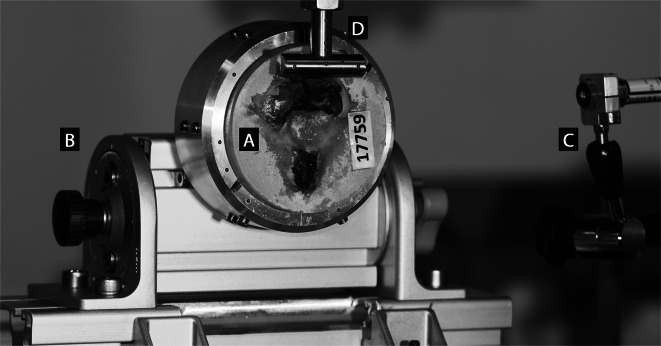
Experimental setup with a specimen mounted for biomechanical testing. The embedded specimen (**A**) was securely placed within an aluminum cup and could be rotated at either − 50° or 0° in the transverse plane. The setup also allowed rotation in the sagittal plane (-15°–0°–15°) using a lockable turning knuckle (**B**). To ensure accuracy, line laser beams (**C**) were used to align the position of the loading cylinder axis (**D**) with the midportion of the odontoid’s articular surface

Biomechanical testing was performed according to a previously described setup (Fig. 4) and protocol [12]. For this purpose, the embedding of each specimen was secured into an experimental setup mounted on the base of an electrodynamic material testing machine (Acumen 3; MTS Systems Corporation, Eden Prairie, MN, USA). The setup allowed for accurate specimen rotation in the sagittal plane determining the vertical load angle (VLA) in three positions, namely − 15° flexion, 0° neutral position, and 15° extension. Rotation in the transverse plane was achieved utilizing line laser beams, defining the horizontal load angle (HLA) at either 0° corresponding to neutral position, or at -50°, representing head rotation to the left. The aforementioned ranges of rotations were chosen to cover the full range of physiological load directions, as previously described [12]. A compressive load was applied to the midportion of the odontoid process’ articular surface at a rate of 0.1 mm/s until catastrophic specimen failure. The load was recorded via a 3 kN load cell (MTS Systems Corporation, Eden Prairie, MN, USA). Load and displacement were digitized at a sample rate of 128 Hz. Fracture-type analysis was performed by a trauma surgeon via visual inspection, and if necessary, using X-rays imaging. Specimens with Anderson d’Alonzo type II and III fracture were fixated with a surgical screw, mounted into the biomechanical setup with the same alignment as during testing in their intact state, and loaded to failure following the same testing protocol.

Based on acquired machine data, the following outcome variables were assessed from the load-displacement curves: stiffness (S, resistance against deformation), yield load (F_YL_, onset of irreversible behavior or damage), and ultimate load (F_UL_, maximum load that the specimen can sustain). Stiffness was calculated from the most linear portion of the load-displacement curve starting at the end of the toe region (corresponding to 1 mm initial displacement) and ending at the highest load level before inducing plasticity, with a local linear regression analysis providing the highest Pearson correlation coefficient over a range of 1 mm displacement with a moving analysis kernel. Yield load was defined as the point where the correlation coefficient of the linear regression decreased below 0.85. Ultimate load was defined as the absolute maximum of the load-displacement curve. The variables were assessed for both the intact state and the instrumented states. To differentiate between these conditions, the variable names were labelled with subscripts ‘int’ and ‘instr’ for intact and instrumented states, respectively. All analyses were performed with in-house Python script and load-displacement curves, and the computed variables were verified visually. In case, a location for outcome assessment was not plausible (e.g., stiffness assessed from a strong non-linear curve or ultimate load in a steadily increasing curve with no major drop in load), manual validation was performed by a senior biomedical engineer (EB). Consequently, stiffness values for 3 and 3 specimens, yield load values for 3 and 5 specimens, and ultimate load values for 4 and 3 specimens from the cannulated screw and allograft bone graft group, respectively, were excluded from further data analyses.

To quantify the relative stabilizing effect of each fixation, the difference between the results of the intact and instrumented states (ΔS, ΔF_YL_, and ΔF_UL_), normalized by the load magnitude in the intact state, F_UL.int_ (baseline condition) was calculated. The result was then subtracted from 1 (100%), to obtain the decrease, compared to the intact state, resulting in the primary outcomes of interest, namely:

$$\eqalign{& {\rm{ the percentage of stiffness restored or }} \cr& \left( {1 - {{\Delta {\rm{s}}} \over {{s_{{\rm{int }}}}}}} \right) \times 100\% \cr} $$

$$\eqalign{& {\rm{ the percentage of yield load restored or}} \cr& {\rm{ }}\left( {1 - {{\Delta {F_{YL}}} \over {{F_{YL.int}}}}} \right) \times 100\% \cr} $$

and.

$$\eqalign{& {\rm{ the percentage of ultimate load restored or }} \cr& \left( {1 - {{\Delta {F_{UL}}} \over {{F_{UL{\rm{.int }}}}}}} \right) \times 100\% \cr} $$

### Surgical procedure

**Fig. 5:**
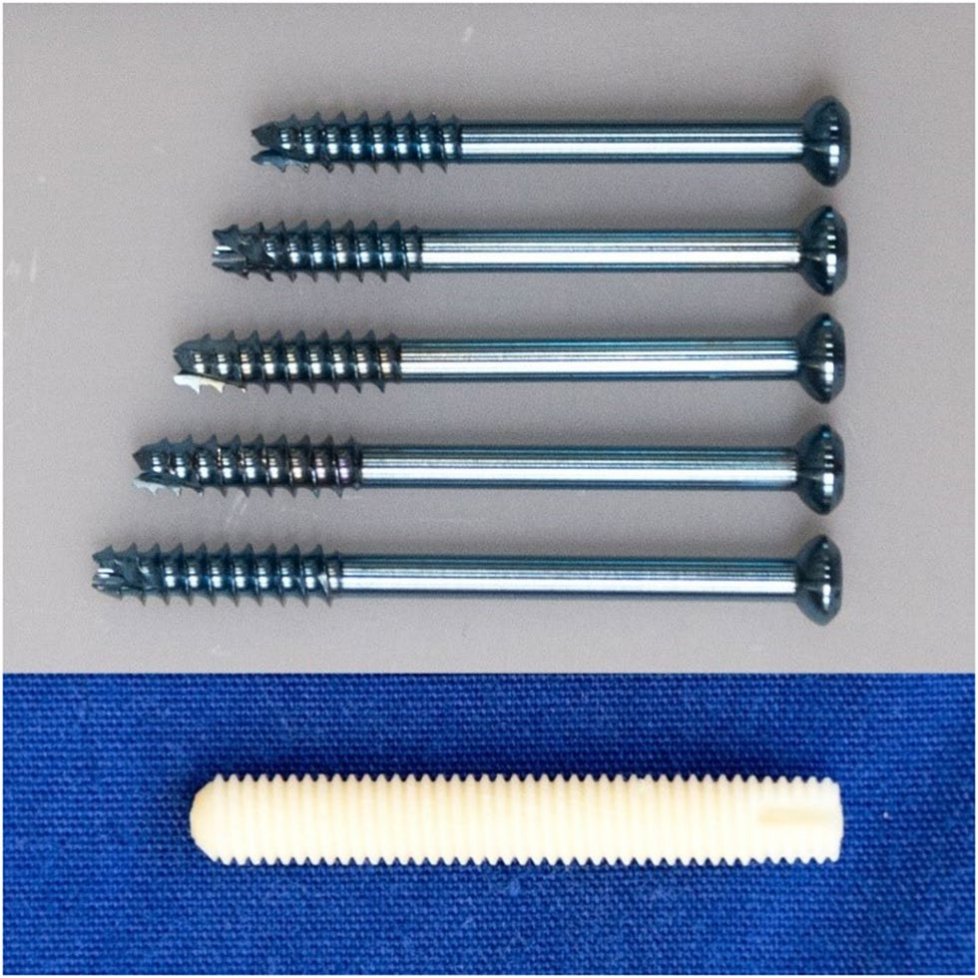
Tested fixation devices (from top to bottom): self-drilling cannulated screws ⌀3.5 mm, L32/12 mm, L34/12 mm, L36/12 mm, L38/12 mm, L40/12 mm (Synthes GmbH, Zuchwil, Switzerland) and, 35 × 5.0 mm Shark Screw Diver (surgebright GmbH, Lichtenberg bei Linz, Austria)

Anterior screw fixation of the odontoid process was performed in both groups. In the cannulated screws group two ⌀3.5 mm self-drilling cannulated screws (Synthes GmbH, Zuchwil, Switzerland) were used. Anatomical reduction of the fragments was performed freehand. The fracture fragments were temporarily fixated with two 200 mm long ⌀1.25 mm guide wires placed from the entry point at the anterior base of the C2 endplate, left and right of the midline, and converging to the tip of the odontoid process. In the sagittal plane, the guide wires were angled slightly posteriorly so that the screws exited at the posterior half of the odontoid’s tip. The position of the guide wires was evaluated visually and under X-rays and repositioned if necessary. A ⌀2.7 mm cannulated drill was used to drill the pilot hole for the screws following the trajectories of the guide wires. The length of the tunnels was measured with a screw length indicator and the screws length was chosen 1–2 mm below the measured length to avoid screw perforation. Cannulated screws were implanted pairing following lengths: 2 × L32/12 mm (*n* = 3), L32/12 mm + L34/12 mm (*n* = 2), 2 × L34/12 mm (*n* = 6), L34/12 mm + L36/12 mm (*n* = 2), 2 × L36/12 mm (*n* = 6), L36/12 mm + L38/12 mm (*n* = 2), 2 × L38/12 mm (*n* = 3), 2 × L40/12 mm (*n* = 1) (Fig. 5). The screws were inserted over the guide wires that were removed afterwards. The final position of the screws was controlled under X-rays.

In the bone allograft screw group, the reduction was performed by hand and a single 200 mm long ⌀1.25 mm guide wire was placed in the sagittal plane from the entry point of the anterior base of the C2 endplate to the tip of the odontoid process. A ⌀2.85 mm drill, followed by a ⌀5.0 mm thread cutter was advanced over the guide wire. A single 35 mm long ⌀5.0 mm threaded bone allograft screw, Shark Screw Diver (surgebright GmbH, Lichtenberg bei Linz, Austria) (Fig.  5) was inserted. Correct implant placement was also controlled under X-rays. Any prominent part of the graft was shortened using an oscillating saw, if necessary.

### Statistical analyses

One-way analysis of covariance (ANCOVA) was conducted to detect significant differences between the two fixations, independent of load angles, regarding the primary outcomes of interest, namely the percentage of stiffness, yield, and peak load restored. Due to a significant difference in donor age (assessed with Mann-Whitney U test) and the resulting differences in vBMD between the two fixation groups, the outcomes were adjusted for the following covariates: stiffness and loads obtained from experiments on specimens in their intact state ($$\:{S}_{int}$$$$\:{F}_{YL.int}$$$$\:{F}_{UL.int}$$, respectively), sex, vBMD of the odontoid process, and the cross-sectional ratio of cortical bone to total bone. Prior to ANCOVA, the homogeneity of regression slopes was evaluated and confirmed no significant interaction of the covariates with each independent variable.

Linear regressions were calculated between the independent and the outcome variables to investigate possible relationship between the overall bone quality and biomechanical stability.

A subgroup analysis was conducted within the bone allograft screw group to compare $\:{F}_{UL.int}$, and $\:{F}_{UL.instr}$ using the Mann-Whitney U test. This analysis aimed to distinguish between specimens that exhibited implant breakage as the mode of failure and those that demonstrated other failure mechanisms.

An α level of < 0.05 was set for all statistical tests. Statistical analysis was performed using IBM SPSS software package (version 27; IBM Corp., Armonk, NY, USA).

## Results

The vBMD and Ct.Ar/Tt.Ar were not significantly different between the two groups (*p* > 0.450, Table 2).

**Table 2:** Descriptive summary of radiological data expressed as mean ± sd (MIN– MAX). Abbreviations: vBMD: volumetric bone mineral density, Ct.Ar/Tt.Ar: cross-sectional ratio of cortical bone to total bone

	vBMD (mg HA/cm³)	Ct.Ar/Tt.Ar (%)
Cannulated screws	*n* = 25 531.40 ± 139.27 (339.91–879.64)	*n* = 24 39.60 ± 9.98 (21.81–64.05)
Bone allograft screw	*n* = 19 556.59 ± 115.87 (371.40–843.29)	*n* = 19 41.88 ± 9.13 (28.84–58.12)

**Fig. 6:**
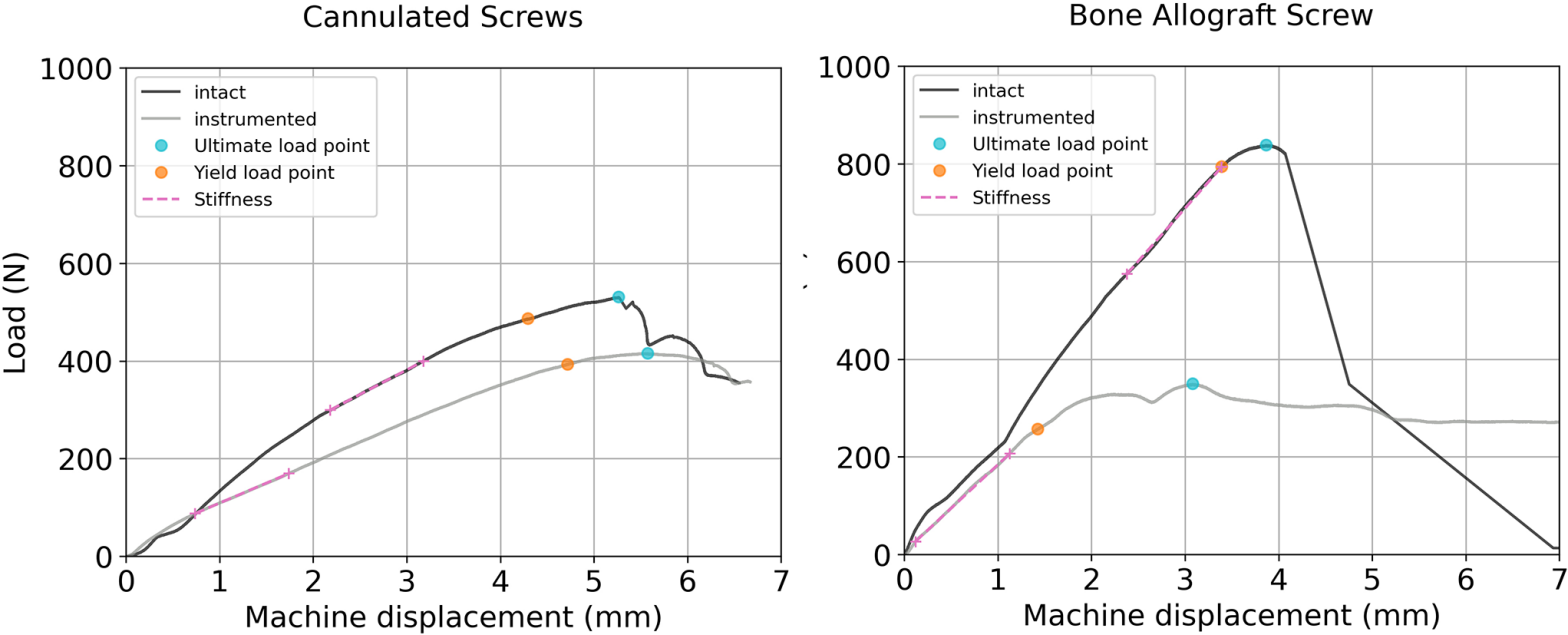
Typical load-displacement curves from both implant groups including the evaluated biomechanical variables

All mechanical tests were completed successfully. However, one specimen was excluded due to incorrect alignment in the test setup. Typical load-displacement curves are presented in Fig. 6 and the experimental outcomes are summarized in Table 3.

**Table 3:** Descriptive summary of experimental outcome data, expressed as mean ± sd (MIN– MAX)

	Stiffness, S (*N*/mm)		Yield load, F_YL_ (*N*)		Ultimate load, F_UL_ (*N*)	
	Intact	Instrumented	Intact	Instrumented	Intact	Instrumented
Cannulated screws	181.3 ± 82.4 (55.7–331.5)	104.9 ± 96.6 (18.4–411.2)	755.4 ± 311.2 (348.1–1358.0)	360.8 ± 219.6 (41.0–950.4)	825.4 ± 340.8 (358.6–1532.7)	452.2 ± 241.6 (87.0–1088.1)
Bone allograft screw	375.8 ± 139.7 (152.3–584.1)	115.5 ± 71.4 (23.3–269.9)	991.0 ± 267.5 (616.8–1329.1)	273.1 ± 140.8 (113.2–528.0)	1130.5 ± 287.9 (751.4–1597.1)	344.8 ± 173.0 (128.3–663.7)

**Fig. 7:**
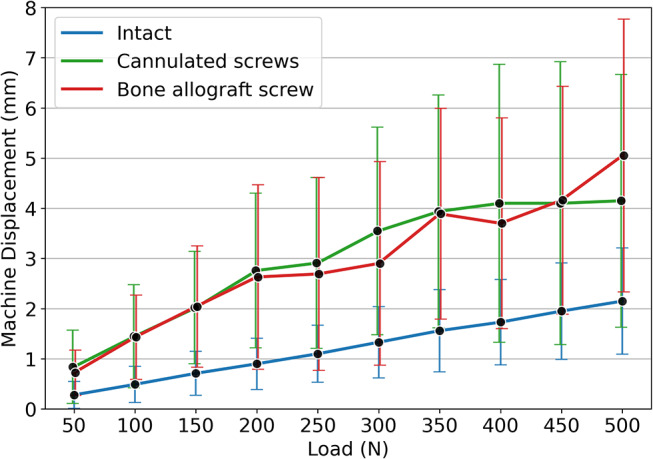
Mean machine displacements over the corresponding load levels for the intact specimens and the following instrumentation with the two implant types. The whiskers represent the standard deviation

The machine displacements were similar between the instrumented groups at defined load levels (Fig. 7).

The mean percentages of stiffness, yield-, and ultimate load restored were calculated (unadjusted model) and adjusted to the mechanical variables of the intact state (S_int_, F_YL.int_, F_UL.int_), sex, age, vBMD and the Ct.Ar/Tt.Ar as presented in Table 4.

**Table 4:** Adjusted and unadjusted mean of the outcome variables with adjustment to the mechanical variables of the intact state (S_int_, F_YL.int_, F_UL.int_), sex, age, vBMD, and the cross-sectional ratio of cortical bone to total bone of the odontoid process. Note that the total number of specimens in each group included in the analyses resulted from exclusion of specimens with corrupt imaging or unplausible mechanical data

		Perc. stiffness restored $\:(1-\frac{{\Delta\:}\text{S}}{{S}_{int}})\times\:100\%$	Perc. yield load restored $\:(1-\frac{{\Delta\:}{F}_{YL}}{{F}_{YL.int}})\times\:100\%$	Perc. ultimate load restored $\:(1-\frac{{\Delta\:}{F}_{UL}}{{F}_{UL.int}})\times\:100\%$
Cannulated screws		*n* = 21	*n* = 22	*n* = 20
	Unadjusted mean ± SD	56.97 ± 43.10	48.08 ± 30.43	49.99 ± 20.08
	Adjusted mean ± SE	44.10 ± 9.51	46.07 ± 6.83	46.14 ± 4.99
Bone allograft screw		*n* = 16	*n* = 15	*n* = 16
	Unadjusted mean ± SD	32.00 ± 22.31	26.84 ± 15.28	28.99 ± 14.67
	Adjusted mean ± SE	49.89 ± 11.59	29.78 ± 8.89	33.81 ± 5.82

There were no statistically significant differences in percentages of stiffness, yield, and ultimate load restored between the two implant groups after adjustment by the mechanical variables of the intact state ($\:S$, $\:{F}_{YL.int}$, $\:{F}_{UL.int}$), sex, vBMD of the odontoid process and the cross-sectional ratio of cortical bone to total bone: F(1,30) = 0.104, *p* = 0.750, F(1,30) = 1.549, *p* = 0.223 and F(1,29) = 1.1903, *p* = 0.178, respectively.

**Fig. 8:**
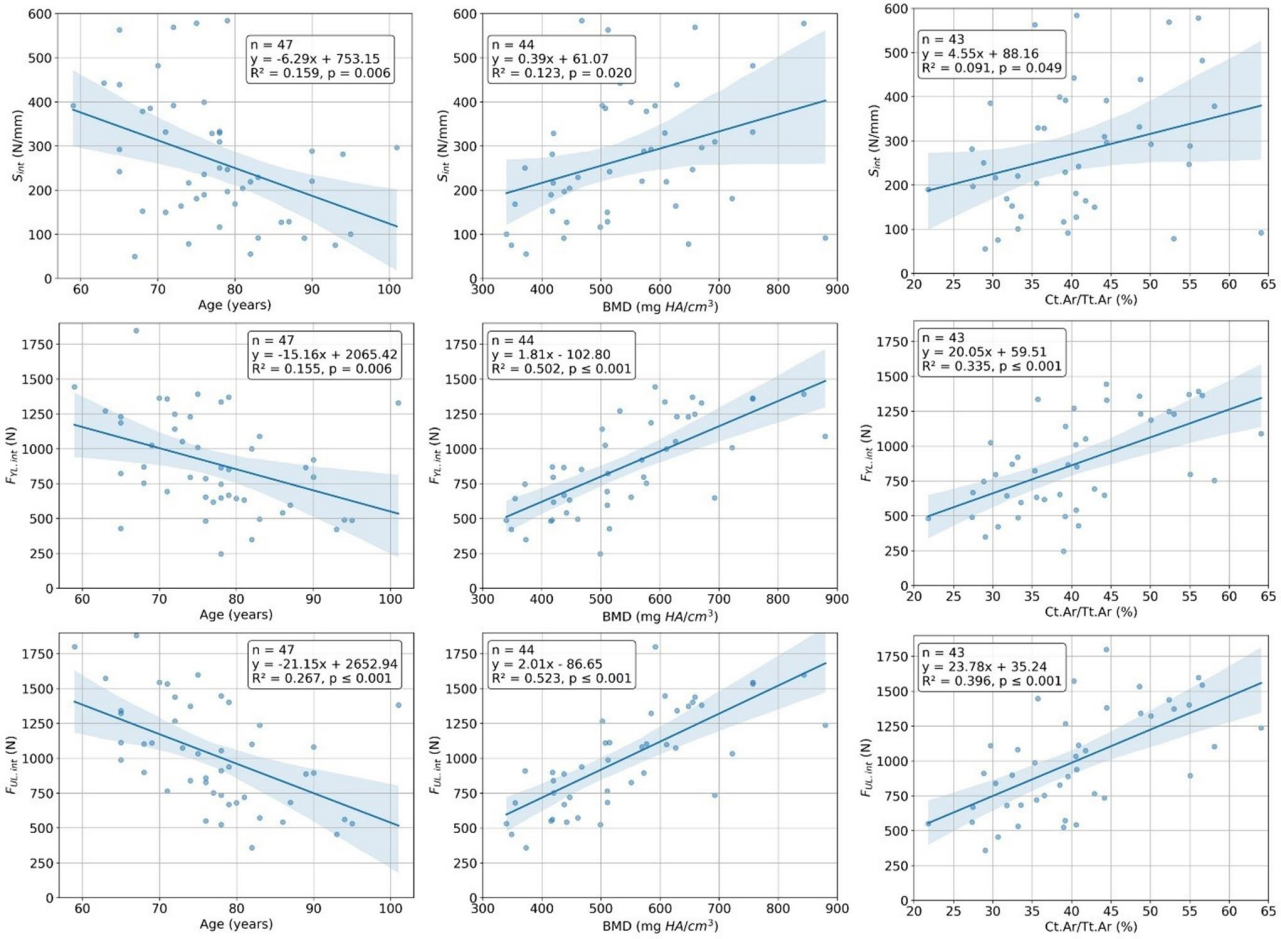
Results from the linear regression analyses between the independent variables age, volumetric bone mineral density of the odontoid process (vBMD), and cross-sectional ratio of cortical bone to total bone in the odontoid process (Ct.Ar/Tt.Ar) and the outcome variables stiffness (S_int_), yield load (F_YL.int_), and ultimate load (F_UL.int_) in the intact state. The scatter plots show the relationship between the variable pairs, along with a fitted regression line and its associated confidence intervals

**Fig. 9:**
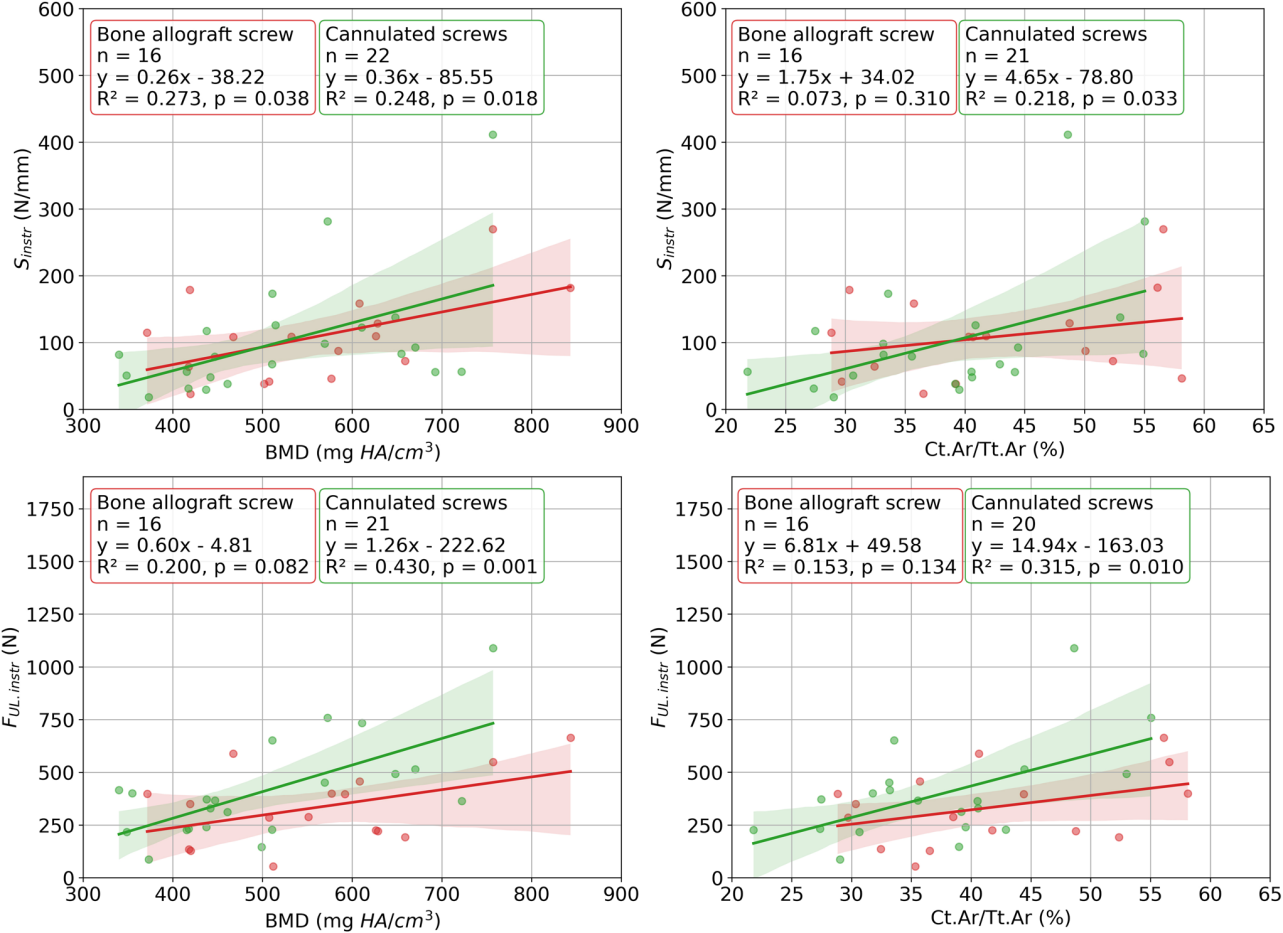
Statistically significant results of the linear regression analyses between independent variables: bone mineral density of the odontoid process (vBMD) and cross-sectional ratio of cortical bone to total bone in the odontoid process (Ct.Ar/Tt.Ar) and outcome variables: stiffness (S_int_) and ultimate load (F_UL.int_) in the instrumented state for both fixation groups. The scatter plots show the relationship between each two variables, along with a fitted regression line and its associated confidence intervals

Each linear regression analysis of the data from the intact specimens tests revealed a statistically significant relation (Fig. 8). The highest, but still moderate correlation was observed between vBMD and F_ult.int_ (R² = 0.523) and the weakest between Ct.Ar/Tt.Ar and S_int_ (R² = 0.091). For instrumented specimens, age did not correlate with any of the outcome variables (0.136 ≤ *p* ≤ 0.747) and none of the independent variables correlated with the F_YL.instr_ (0.121 ≤ *p* ≤ 0.418). All significant correlations are visualized in Fig. 9.

There was a positive and statistically significant relation between the yield and ultimate load for the intact (R² = 0.900, *p* < 0.001) bone allograft screw (R² = 0.880, *p* < 0.001) and cannulated screws group (R² = 0.889, *p* < 0.001).

In the cannulated screws group, there were 8 (36%) midsection and 3 (12%) vertebral body fractures. In 12 (24%) and 2 (8%) specimens, the screws cut-out in the caudal and cranial region, respectively. No mechanical damage to the cannulated screws was observed. In the bone allograft screw group, the graft broke in 7 specimens (33%). In 2 (10%) and 6 (29%) specimens, the graft loosened in the cranial and caudal region, respectively. Cranial and caudal graft cut-out was observed in 4 (19%) and 1 (5%) case, respectively. In one specimen (5%), it was not feasible to clearly define the exact failure mechanism. In the bone allograft group, specimens experiencing implant breakage exhibited significantly higher ultimate loads both in the intact state (1443.6 N vs. 1096.5 N, *p* = 0.038) and the instrumented state (473.8 N vs. 250.4 N, *p* = 0.011) compared to specimens with failure mechanisms other than implant breakage.

## Discussion

Despite the high incidence of odontoid fracture in the elderly, their fixation is still associated with a number of complications following treatment, including non-union in 8.1% [5] of cases and death specifically 14% within 30 days of presentation [4]. Treatment using one or two metal screws remains the standard surgical treatment. This study aimed to compare the biomechanical competence of commercially available bone allograft screws to restore the physiological stability of the odontoid process. With a total of 47 surgically treated specimens, tested within the full physiological range of loading and considering the local bone geometry and density, this is– by far– the most extensive biomechanical study on odontoid fracture fixation.

The model, adjusted by mechanical variables of the intact state (S_int_, F_YL.int_, F_UL.int_), sex, age, vBMD of the odontoid process and the cross-sectional ratio of cortical bone to total bone did not reveal inferior ability of the bone allograft screw in restoring the physiological stability.

One of the most important findings of this study is that the bone allograft fixation provided similar stiffness as two cannulated screws. Maximizing construct rigidity is the purpose of odontoid fracture fixation to enable primary bone healing. By ensuring comparable fixation stiffness and expectedly improved biological support, the bone allograft is expected to lead to improved healing outcomes. The stiffness restored in both fixation groups was approximately half of the physiological stiffness of the intact state. The stiffness measured in instrumented specimens encompasses the combined rigidity of the bone-implant construct. Thus, the restored stiffness in the cannulated screw group could be expected to be higher due to the significantly greater stiffness of the metal implants compared to bone material of allograft screw. The opposite result indicates that the most compliant part on the bone-implant construct is the trabecular bone hosting the implant. The statistically significant correlations, especially of vBMD with the mechanical variables further underlines this finding.

The ultimate load, describing the catastrophic fixation failure, was approximately 50% higher in the intact state compared to the instrumented specimens due to brittleness of bone tissue. Yield load marks the onset of irreversible deformation or mechanical damage. While the ultimate load is clearly identifiable as the maximum load value in the load-displacement curves, the yield load was calculated as a non-linearity threshold in the curves. Nevertheless, the high correlation between the yield and ultimate loads for the intact and instrumented specimens (0.880 ≤ R² ≤ 0.900, *p* < 0.001) confirmed the reliability of the proposed methodology. The difference in the yield load between the intact and instrumented specimens was more prominent than for ultimate load. The reason is likely the gradual destruction of the soft trabecular bone hosting the implants, occurring at lower load levels before the ultimate load is reached often involving the destruction of the hard cortical bone. Even though, there was no statistically significant difference in the restored yield nor ultimate load between the two fixation groups, although the cannulated screws group demonstrated higher mean values. The bone allograft screw restored approximately one third of the physiological yield and ultimate loads. The proposed consequence in patients treated with the bone allograft screws would be more conservative post-surgical treatment. Especially, the risk of falls should be prophylactically minimized, e.g., with walking assistance or walking aids, to reduce the magnitude of peak loads that could inhibit fracture healing or cause graft breakage.

In the cannulated screws group, no mechanical implant damage was observed, while one-third of the bone allograft screws broke. Although implant breakage is considered an unfavorable clinical outcome, it occurred in the bone allograft screw group in specimens that reached significantly higher ultimate loads in both the intact and instrumented states. This leads to the conclusion that in those specimens the bone was stronger than the bone allograft screws. Thus, the failure was determined by the implant.

In a case report, histological and ultrastructural investigations have shown the formation of new vessels within the Haversian canals of the Shark Screw bone allograft, as well as bone remodeling at the host bone-allograft interface and within the allograft itself [10]. It is hypothesized that using bone allograft screws could enhance fracture healing and improve primary fixation strength over time. Given the fracture fusion is observed in over 90% of patients ≤ 70 years, in contrast to only in 63% of patients > 70 years [13], the use of bone allograft screws would be especially beneficial for older patients.

In the present study, a double-screw construct was used, while the optimal number of inserted screws in C2 vertebra is an ongoing debate. The second screw might offer the theoretical advantage of preventing rotation between the odontoid process relative to the body of C2 [11]. The discussion on the optimal number of screws is often based on the results of two existing biomechanical studies [14, 15] that included small sample sizes or the odontoid fracture was created by its osteotomy resulting in clinically atypical fracture surfaces. Some larger clinical studies [16, 17] found higher union rates in patients treated with the double-screw, compared to the one-screw technique. Lvov et al. [5] conducted a systematic review and meta-analysis of clinical studies on odontoid fracture fixations using screws published between 1982 and 2019. The only significant difference found between the single and double-screw technique was a higher cut-out rate in patients aged 65 and older who underwent double-screw fixation. The authors argued that this could be due to the beak-shaped anterior part of the C2. Consequently, screw trajectories are positioned more superficially when using two screws compared to one screw, which could result in earlier biomechanical instability in older patients with inferior bone quality. However, the data from the present study did not align with this assumption. In the cannulated screws group, the specimens that failed due to caudal screw cut-out (*n* = 12, 24%) showed a mean vBMD of 516.1 mg HA/cm³ and mean ultimate load of 884.8 N, while the mean vBMD and ultimate load of this group was 531.4 mg HA/cm³ and 825.4 N, respectively.

This study has three major limitations. First, anatomic specimens are generally not unlimitedly available and donors are rarely young or middle-aged. Thus, the present study was conducted on a limited number of specimens from older donors. Second, the specimens of the cannulated screws group belonged to older donors than the specimens of the bone allograft screw group. Consequently, this was reflected in lower inferior bone quality as well biomechanical performance. Both aforementioned limitations were addressed by compensating for the specimen-specific sex, age, vBMD and Ct.Ar/Tt.Ar, as well as the biomechanical quantities of intact specimens in the statistical analysis. Third, the applied unidirectional quasistatic loading does not replicate in vivo conditions. I*n vivo* conditions of traumatic events are often largely unknown and difficult to simulate in vitro. The applied quasi-static loading rate was selected to facilitate better control over the experiments. 80% of odontoid fractures in patients older than 70 years are attributed to low-energy falls, whereas the majority of fractures in younger patients is due to high-energy trauma (i.e., road traffic accidents, falls from significant height) [3, 13], justifying, to a certain extent, the low loading rate for the used specimens of older donors. In order to quantify the stabilizing ability of each fixation as a ratio to the physiological stability, a load-to-failure test was performed. This prevents drawing of any conclusion on progressive implant loosening. Simultaneously, it is not feasible to simulate any pathophysiological or biological processes that would likely have influenced the overall stability of the different fixations over time. Therefore, all conclusions of this study are limited to their primary stabilizing ability of the fixations.

## Conclusion

The bone allograft screw demonstrated comparable biomechanical competence in odontoid fracture type II and III fixation when compared to use of two cannulated screws, with no statistically significant inferiority observed. Its biointegrative properties and other clinical advantages make it a promising treatment option, especially for older patients, provided that they undergo a more conservative post-surgical treatment and close monitoring of fracture site healing. Nevertheless, additional clinical studies with sufficient sample sizes are needed to validate these findings.

## Data Availability

The data that support the findings of this study are not openly available due to reasons of sensitivity and are available from the corresponding author upon reasonable request.

## Declarations

### Generative AI and AI-assisted technologies in the writing process

During the preparation of this work the authors used ChatGPT 3.5 (OpenAI, Inc., San Francisco, CA, USA) in order to improve readability and language. After using this chatbox, the authors reviewed and edited the content as needed and take full responsibility for the content of the publication.
